# Dietary short-chain fatty acid intake improves the hepatic metabolic condition via FFAR3

**DOI:** 10.1038/s41598-019-53242-x

**Published:** 2019-11-12

**Authors:** Hidenori Shimizu, Yuki Masujima, Chihiro Ushiroda, Rina Mizushima, Satsuki Taira, Ryuji Ohue-Kitano, Ikuo Kimura

**Affiliations:** 1 Department of Applied Biological Science, Graduate School of Agriculture, Tokyo University of Agriculture and Technology, Fuchu-shi, Tokyo, 183-8509 Japan; 2 AMED-CREST, Japan Agency for Medical Research and Development, Chiyoda-ku, Tokyo, 100-0004 Japan; 3 NOSTER Bio-Institute, Nitto Pharmaceutical Industries, Ltd., Kamiueno, Muko, Kyoto, 617-0006 Japan

**Keywords:** Endocrine system and metabolic diseases, Fat metabolism, Nutritional supplements, Molecular biology

## Abstract

Fermented foods represent a significant portion of human diets with several beneficial effects. Foods produced by bacterial fermentation are enriched in short-chain fatty acids (SCFAs), which are functional products of dietary fibers via gut microbial fermentation. In addition to energy sources, SCFAs also act as signaling molecules via G-protein coupled receptors such as FFAR2 and FFAR3. Hence, dietary SCFAs in fermented foods may have a direct influence on metabolic functions. However, the detailed mechanism by dietary SCFAs remains unclear. Here, we show that dietary SCFAs protected against high-fat diet-induced obesity in mice in parallel with increased plasma SCFAs without changing cecal SCFA or gut microbial composition. Dietary SCFAs suppressed hepatic weight and lipid synthesis. These effects were abolished in FFAR3-deficient mice but not FFAR2-deficient. Thus, SCFAs supplementation improved hepatic metabolic functions via FFAR3 without influencing intestinal environment. These findings could help to promote the development of functional foods using SCFAs.

## Introduction

Dietary fiber is considered to be an essential healthy component of diet, and has been demonstrated to reduce the risk of metabolic diseases such as obesity and diabetes^1,2^. These benefits are mainly attributed to the influence of dietary fibers on increasing the levels of short-chain fatty acids (SCFAs), i.e., acetate, propionate, and butyrate, in the colon through gut microbial fermentation^3,4^. Various fermented foods made by bacterial fermentation, including cheese, butter, alcoholic beverages, pickles, sauerkraut, soy sauce, and yoghurt, are also highly enriched in SCFAs^5–7^; vinegar and alcoholic beverages contain acetate, cheese contains propionate and butyrate, and butter contains butyrate^8–11^. Fermented food products have also been reported to enhance nutrition, improve health, and prevent diseases, including cardio-metabolic disease and type 2 diabetes, on a global level^12–14^. However, the detailed metabolic benefits of fermented foods and the underlying mechanisms have not been completely elucidated to date.

SCFAs can not only be used for the *de novo* synthesis of lipids and glucose as the main energy sources for the host^15,16^ but also influence host physiological functions. Hence, SCFAs have been proposed to play an important role in the prevention and treatment of metabolic diseases. Despite some contradictory reports^17,18^, the metabolic benefits of SCFAs are widely accepted and supported with several lines of evidence^19–24^. Indeed, SCFAs were shown to improve metabolic function in mice and humans, demonstrating a direct causal relationship between the fermentation of dietary fibers and SCFAs^25–28^. SCFAs could also protect against diet-induced obesity and insulin resistance via systemic effects^29–31^. However, acetate was also reported to promote obesity via hyperphagia and insulin secretion in rodents^32^, and propionate impaired the action of insulin via glucagon secretion^17^.

Recent studies have shown that these metabolic benefits by SCFAs are exerted via their receptors, such as the G protein-coupled receptor GPR41/FFAR3 and GPR43/FFAR2. They are free fatty acids receptors with Gi/o coupling for FFAR3 and dual coupling through the Gi/o and Gq families for FFAR2. These receptors are activated by SCFAs with EC50 values of μM order^33,34^. Therefore, SCFAs exert not only intestinal effects but also systemic effects through circulating in the plasma^35^. FFAR3 regulates neural activity, glucose homeostasis, and lipid metabolism via secretion of catecholamine, endocrine hormones, and gut hormones^36,37^, whereas FFAR2 regulates glucose homeostasis and lipid metabolism via insulin action, the inflammatory response, and gut hormone secretion^38–40^. With oral administration, nutrients such as SCFAs are almost completely absorbed in the small intestine^25^. However, the detailed mechanisms underlying the difference in the metabolic benefits conferred by the direct intake of dietary SCFAs (such as through fermented foods) and those provided by gut microbes producing SCFAs from dietary fibers in the colon remain unclear.

To clarify these effects, in this study, we investigated the SCFAs-mediated systemic effects via circulating plasma upon dietary SCFA intake in a mouse model of high-fat diet (HFD)-induced obesity. We confirmed that dietary SCFA intake such as fermented foods mainly causes improved hepatic metabolic conditions via FFAR3 in HFD-induced obese mice.

## Results

### Dietary SCFA intake increases plasma SCFA levels

We firstly investigated changes in incorporated SCFAs level following SCFAs feeding in a mouse model of HFD-induced obesity. In this experiment, 7-week-old mice were fed HFDs containing 5% SCFAs (acetate, propionate, and butyrate, respectively), 5% cellulose or normal HFD as control for 4 weeks (Supplementary Table S1). Feeding of diets supplemented with each SCFA significantly increased the corresponding SCFA levels compared with those of the cellulose-supplemented and control HFD-fed mice (Supplementary Fig. S1A), whereas there were no changes in the cecal SCFAs levels (Supplementary Fig. S1B). Moreover, 16S rRNA gene amplicon sequencing confirmed that the SCFA diets did not alter the relative abundance of the major phyla constituting the gut microbiota (Supplementary Fig. S1C) or the gut microbiota composition, as indicated by principal coordinates analysis with reference to taxonomic datasets (Supplementary Fig. S1D).

### Dietary SCFA intake exerts metabolic benefits

**Figure 1:**
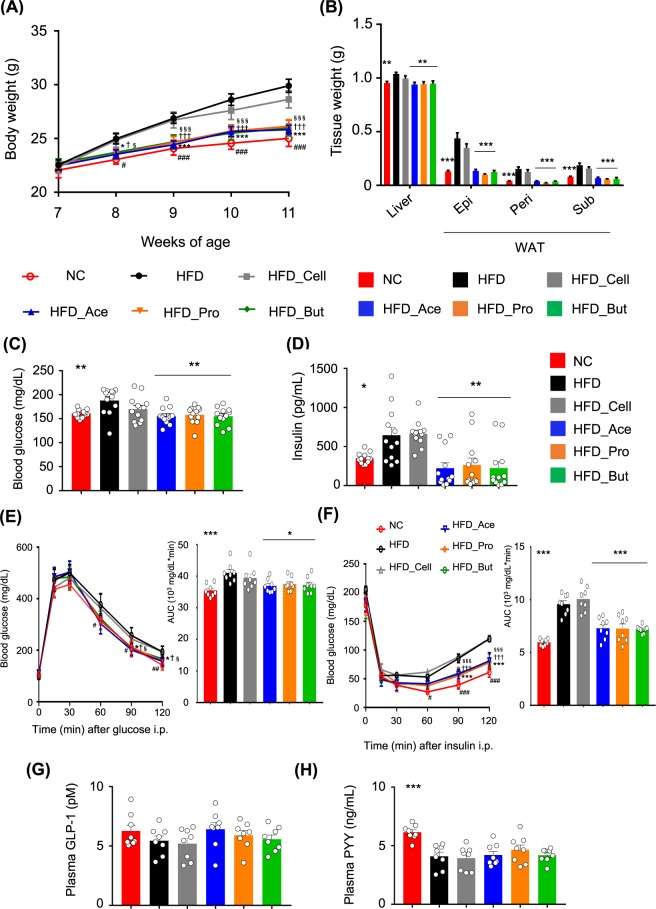
Short-chain fatty acid (SCFA) supplementation exerts metabolic benefits. Body weight changes (**A**), liver and white adipose tissue (WAT) weights (**B**), blood glucose (**C**), plasma insulin (**D**), glucose tolerance test (GTT) (**E**), insulin tolerance test (ITT) (**F**), GLP-1 (**G**), and PYY levels (**H**) measured after 4 weeks of normal chow (NC) or high-fat diet (HFD) feeding supplemented with 5% SCFAs. All data are presented as the means ± SEM (*n* = 8–12). Dunnett’s test; ***P < 0.001, **P < 0.01, and *P < 0.05, compared with HFD. ^###^*P* < 0.001, ^##^*P* < 0.01, and ^#^*P* < 0.05 (HFD vs. NC), ***P < 0.001 and *P < 0.05 (HFD vs. Ace), ^†††^*P* < 0.001 and ^†^*P* < 0.05 (HFD vs. Pro), ^§§§^*P* < 0.001 and ^§^*P* < 0.05 (HFD vs. But) (**A,E,F**). Epi: epididymal tissue, Peri: perirenal tissue, Sub: subcutaneous tissue.

We next investigated changes in metabolic parameters following SCFAs feeding. HFD feeding to adult mice for 4 weeks can sufficiently induce obesity and related metabolic disturbances as compared to that in normal chow-fed mice (Fig. 1A–F)^41–43^. Body weights in the SCFAs-fed mice were significantly lower than those in the cellulose-fed and control mice during growth (Fig. 1A). The white adipose tissue (WAT) mass and liver weights (Fig. 1B), blood glucose levels (Fig. 1C), and plasma insulin levels (Fig. 1D) were also significantly lower in the SCFA groups than those in the cellulose-fed and control mice at 11 weeks of age. Furthermore, HFD-induced insulin resistance and impaired glucose tolerance, as determined by the glucose tolerance test (GTT) and insulin tolerance test (ITT), respectively, were significantly attenuated in SCFA-fed mice as compared to those in cellulose-fed and control mice (Fig. 1E,F). However, the plasma levels of the gut hormones glucagon like peptide-1 (GLP-1) and peptide YY (PYY) were similar among all groups, although plasma GLP-1 tended to be slightly higher in the SCFA-fed groups (Fig. 1G,H). Food intake was also similar among all groups (Supplementary Fig. S2).

### Dietary SCFA intake improves hepatic metabolic conditions

**Figure 2:**
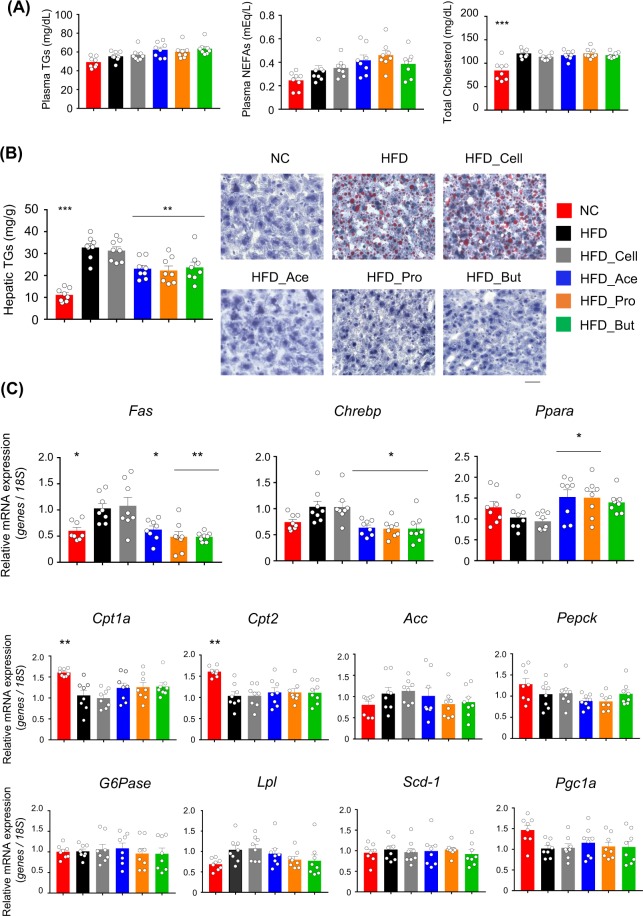
Dietary short-chain fatty acid (SCFA) intake improves hepatic metabolic conditions. Plasma triglycerides, non-esterified fatty acids (NEFAs), and total cholesterol concentrations (**A**), hepatic triglyceride contents and histology of hepatocytes based on hematoxylin-eosin (H&E) oil red O stanning. Scale bar, 50 μm (**B**), and mRNA expression levels of hepatic energy metabolism-related genes (**C**) measured after 4 weeks of normal chow (NC) or high-fat diet (HFD) feeding supplemented with 5% SCFAs. All data are presented as the means ± SEM (*n* = 8). Dunnett’s test; ***P < 0.001, **P < 0.01, and *P < 0.05, compared with HFD.

We further examined changes in lipid metabolism. Although plasma triglycerides (TGs), non-esterified fatty acids (NEFAs), and total cholesterol levels were comparable between each SCFAs-fed group and the cellulose-fed or control group (Fig. 2A), hepatic TG levels in each group of SCFA-fed mice were significantly lower than those in cellulose-fed and control mice (Fig. 2B). We also investigated the expression profiles of hepatic genes related to energy metabolism. The expression levels of *Fas* and *Chrebp—*which are related to fatty acid synthesis—decreased, while that of *Ppara*—as a key regulator of lipid metabolism with acetate and propionate feeding—was increased in the SCFAs-fed mice compared to those of cellulose-fed and control mice (Fig. 2C). However, these changes in genes related to energy metabolism were not observed in the WAT or muscle (Supplementary Figs S3 and 4). Thus, dietary SCFA intake suppressed the HFD-induced accumulation of hepatic TGs via changing hepatic lipid metabolism but not the WAT and muscle metabolism.

### FFAR3 deficiency abolishes the metabolic benefits of dietary SCFA intake

**Figure 3:**
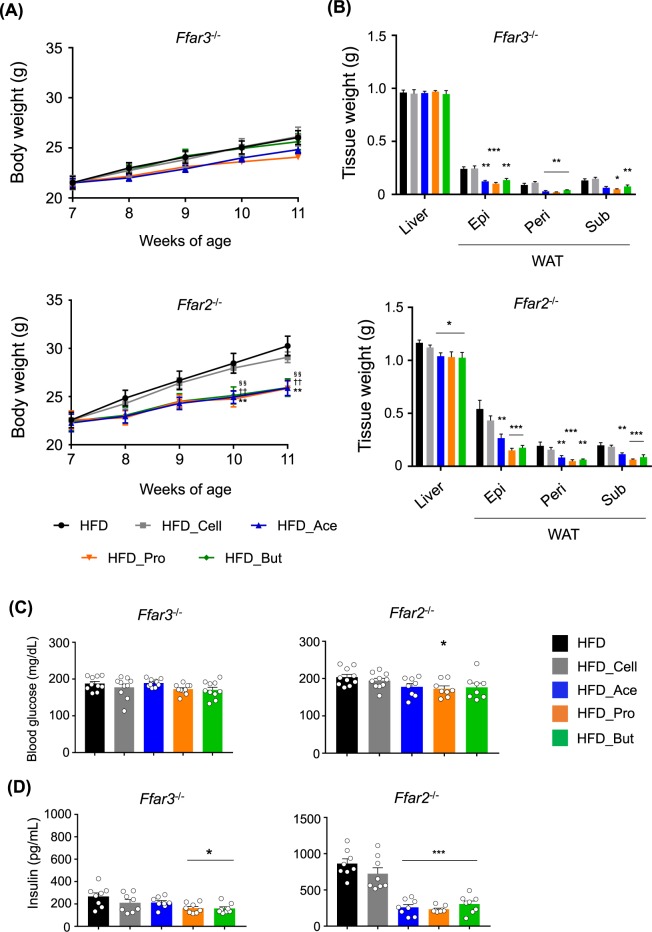
FFAR3 deficiency abolishes dietary short-chain fatty acid (SCFA) intake-induced metabolic benefits. Body weight changes (**A**), liver and white adipose tissue (WAT) weights (**B**), blood glucose (**C**), and plasma insulin (**D**) levels in *Ffar3*^−/−^ (*n* = 10) and *Ffar2*^−/−^ (*n* = 8–10) mice measured after 4 weeks of high-fat diet (HFD) feeding supplemented with 5% SCFAs. All data are presented as the means ± SEM. Dunnett’s test; ***P < 0.001, **P < 0.01, and *P < 0.05, compared with HFD. **P < 0.01 (HFD vs. Ace), ^††^*P* < 0.01 (HFD vs. Pro), ^§§^*P* < 0.01 (HFD vs. But) (**A**). Epi: epididymal tissue, Peri: perirenal tissue, Sub: subcutaneous tissue.

SCFAs exhibit various physiological functions related to energy regulation^13^. Therefore, we next investigated the roles of the SCFA receptors FFAR2 and FFAR3 in the observed metabolic improvement by increased plasma SCFAs from dietary SCFA intake using SCFA receptor-deficient mice. Before HFD feeding, initial body and tissue weights, and blood glucose, as well as hepatic lipid metabolic related genes such as *Fas*, *Chrebp*, and *Ppara* were similar among three groups (Supplementary Fig. S5). Although food intake was comparable among the three groups (Supplementary Fig. S2), the suppression of HFD-induced weight gain by SCFAs supplementation was attenuated in *Ffar3*^−/−^ mice, especially with butyrate feeding, whereas the effects in *Ffar2*^−/−^ mice were comparable with those in wild-type mice (Fig. 3A). Moreover, although the HFD-induced WAT mass increase was suppressed by the SCFAs in both *Ffar3*^−/−^ and *Ffar2*^−/−^ mice, similar to the wild-type mice, the suppression of HFD-induced liver weight gain by SCFAs feeding was abolished in the *Ffar3*^−/−^ mice but not in the *Ffar2*^−/−^ mice (Fig. 3B). The changes in blood glucose by SCFAs feeding were also completely abolished in *Ffar3*^−/−^ mice and were attenuated in *Ffar2*^−/−^ mice (Fig. 3C). Although basal plasma insulin levels in HFD-fed mice were lower in *Ffar3*^−/−^ mice and higher in *Ffar2*^−/−^ mice, the SCFAs suppressed these changes in both *Ffar2*^−/−^ and *Ffar3*^−/−^ mice, similar to the effects observed in wild-type mice (Fig. 3D). These results indicated that the functions of FFAR3 in the liver are partially related to the metabolic benefits from dietary SCFA intake.

### FFAR3 deficiency abolishes the SCFAs-induced expression changes of hepatic lipid metabolism-related genes

**Figure 4:**
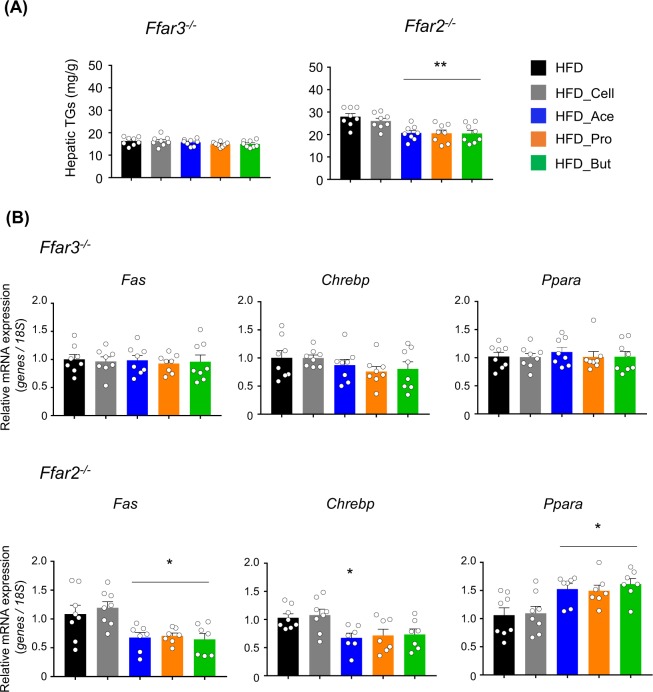
FFAR3 deficiency abolishes dietary short-chain fatty acid (SCFA) intake-induced expression change of hepatic lipid metabolism-related genes. Hepatic triglycerides contents (**A**) and mRNA expression levels of hepatic lipid metabolism-related genes (**B**) in *Ffar3*^−/−^ and *Ffar2*^−/−^ mice (*n* = 7–8) after 4 weeks of high-fat diet (HFD) feeding supplemented with 5% SCFAs. All data are presented as the means ± SEM. Dunnett’s test; **P < 0.01 and *P < 0.05, compared with HFD.

We next examined the influence of SCFAs on liver function in the SCFA receptor-deficient mice. Although the basal hepatic TG levels in HFD-fed mice were relatively lower in *Ffar3*^−/−^ mice, SCFAs feeding did not suppress hepatic TG accumulation, whereas this increase was suppressed in *Ffar2*^−/−^ mice as well as in wild-type mice (Fig. 4A). Moreover, the SCFAs-induced changes in hepatic lipid metabolism-related genes such as *Fas*, *Chrebp*, and *Ppara* were also abolished in *Ffar3*^−/−^ mice but not in *Ffar2*^−/−^ mice (Fig. 4B). Thus, dietary SCFA intake improves hepatic lipid metabolism via FFAR3.

**Figure 5:**
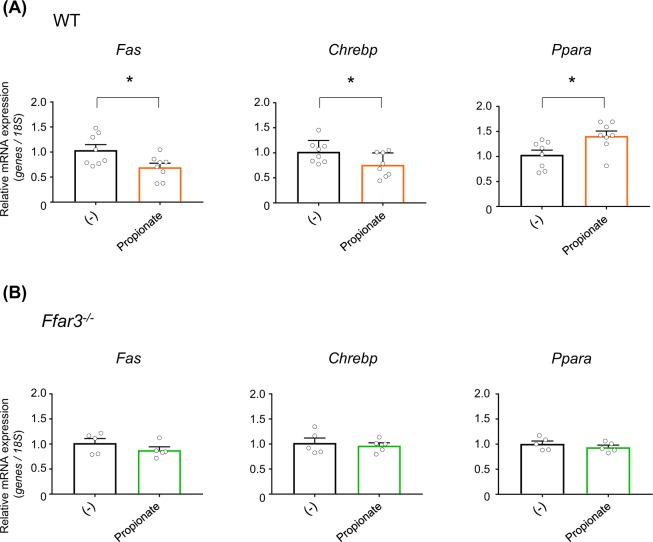
FFAR3 deficiency abolishes acute short-chain fatty acid (SCFA) injection-induced expression changes in hepatic lipid metabolism-related genes. mRNA expression levels of hepatic lipid metabolism-related genes in wild-type (WT) (*n* = 8) (**A**) and *Ffar3*^−/−^ (**B**) mice (*n* = 5) at 24 h after intraperitoneal phosphate buffered saline (for control group) or propionate administration (1 g/kg body weight) under high-fat diet (HFD) feeding. All data are presented as the means ± SEM. Student’s *t*-test; *P < 0.05, compared with (−).

Finally, we investigated whether SCFAs influence hepatic lipid metabolism under direct stimulation rather than as a secondary long-term effect. Intraperitoneal administration of propionate, as the most potent agonist for FFAR3, transiently increases plasma propionate levels^37^. Similar to the effects of long-term dietary SCFAs feeding, acute propionate injection decreased the expression levels of hepatic *Fas* and *Chrebp*, and increased the expression level of hepatic *Ppara* in wild-type mice (Fig. 5A) without changing the body weight, liver weight, blood glucose, plasma TGs, and hepatic TGs (Supplementary Fig. S6). By contrast, the effects of acute propionate administration were almost completely abolished in *Ffar3*^−/−^ mice (Fig. 5B). These results confirmed that dietary SCFA intake improves hepatic metabolic conditions via plasma SCFA-stimulated FFAR3.

## Discussion

Dietary fiber and its gut microbial metabolite SCFAs are well known to exert metabolic benefits to the host, and the mechanisms have been extensively examined. However, the underlying mechanism of the metabolic benefits provided by dietary SCFA intake, such as with fermented food enriched in SCFAs, are less well understood. Here, we demonstrate that dietary SCFA intake increases plasma SCFA levels to active FFAR3 and improves hepatic metabolic conditions without changing the intestinal environment.

Indeed, dietary SCFA intake increased plasma SCFAs levels but not cecal SCFAs levels without changing the gut microbial compositions in HFD-fed mice. Consequently, dietary SCFA intake dramatically suppressed the HFD-induced liver and WAT weight gains, without influencing gut hormones. This result indicates that direct intake of dietary SCFAs such as consumption of fermented foods also exhibits metabolic benefits. Although these effects appear to be similar to those of dietary fiber, the mechanism may differ to some degree. For example, dietary SCFA intake did not sufficiently change plasma gut hormone levels, although plasma GLP-1 tended to be slightly higher in the SCFA-fed groups, because almost all SCFAs are absorbed in the small intestine and they are not readily transfered to the colon^25^, whereas dietary fiber intake produces SCFAs via fermentation by gut microbiota mainly in the colon. L-cells producing GLP-1 and PYY are localize mainly in the distal ileum and colon. Therefore, direct SCFA intake might mainly exert systemic effects via the plasma SCFAs rather than via distal intestinal SCFAs. Further investigation is needed to test this hypothesis and clarify the difference between the effects of direct SCFA intake and gut microbiota-produced SCFAs.

We found that dietary SCFA intake suppressed the HFD-induced liver weight gain and hepatic TGs accumulation along with a change in hepatic lipid metabolism-related genes, and these effects were abolished by FFAR3 deficiency but not FFAR2 deficiency. Similarly, a previous study showed that dietary SCFA intake suppressed the synthesis of hepatic fatty acids^44^. Hence, we concluded that dietary SCFA intake improves hepatic metabolic conditions via FFAR3. However, since FFAR3 is barely expressed in the liver^36^, it is more likely that plasma SCFAs indirectly influence hepatic lipid metabolic-related genes such as *Fas* and *Ppara* in the liver via other FFAR3-expressing tissues. Given the high expression of FFAR3 in the peripheral nerves^36^, FFAR3-mediated neural activity might explain the observed improvement of hepatic metabolic conditions by SCFAs. The FFAR3-FFAR2 heteromer and species difference influence different intracellular signaling pathways^45,46^ and therefore, it might also exert other physiological effects. To further clarify this FFAR3-mediated molecular mechanism, further experiments are needed with tissue-specific FFAR3-deficient mice.

SCFAs are known to influence insulin actions via their receptors^47,48^. Indeed, the plasma insulin levels in HFD-fed control and cellulose-supplemented mice were already drastically lower at baseline in *Ffar3*^−/−^ mice compared with those of wild-type mice. Hence, this difference might partially explain why the protection of dietary SCFA intake against HFD-induced obesity and hyperglycaemia was abolished in the *Ffar3*^−/−^ mice. However, acute SCFAs intraperitoneal administration in *Ffar3*^−/−^ mice also abolished the changes of SCFAs-induced hepatic lipid metabolism-related genes observed in wild-type mice. Accordingly, improvement of hepatic metabolic conditions by SCFAs appears to be due, at least in part, to the direct effects via FFAR3. Moreover, the protective effect of dietary SCFA intake against HFD-induced WAT weight gain was not abolished in both *Ffar3*^−/−^ and *Ffar2*^−/−^ mice. This indicates that other SCFA receptors beside FFAR2 and FFAR3, SCFA-mediated bioactivities, or the confounding effects of interactions between FFAR2 and FFAR3 might be related to the SCFA-mediated suppression of adipose fat accumulation. To further clarify this SCFA and receptor-mediated molecular mechanism, further experiments are also needed with FFAR2/FFAR3-double-deficient mice.

Overall, we showed that dietary intake of the three major SCFAs, acetate, propionate, and butyrate, protected against HFD-induced obesity, and improved hepatic metabolic conditions via FFAR3 in mice. These findings demonstrate that SCFAs themselves, in addition to the dietary fiber and/or gut microbiota, have anti-obesity effects and could be used to improve metabolic conditions. Thus, we have provided novel insight into the mechanism underlying the beneficial effects of fermented foods. Accordingly, these results may guide the development of functional foods for the prevention of metabolic disorders such as obesity and type 2 diabetes mellitus.

## Methods

### Mice, diet, and experimental design

Male C57BL/6J mice were purchased from Japan SLC (Shizuoka, Japan) and maintained under a strict 12-h light/dark cycle in a conventional animal room at 23.0 °C and 40–70% relative humidity. The mice were acclimated to the laboratory conditions on the CLEA Rodent Diet (CE-2, CLEA Japan, Inc., Tokyo, Japan) for 1 week prior to the treatment. The 7-week-old C57BL/6J mice were placed on a D12492 diet (HFD: 60% of calories from fat; Research diets, New Brunswick, NJ, USA) or an HFD containing 5% cellulose, acetate, propionate, or butyrate for 4 weeks (*n* = 12 per group). The compositions of the diets are given in Supplementary Table S1. These diets were adjusted so that the final percentages of protein, fat, and carbohydrates were almost equal in all groups. In all experiments, the 7-week-old mice were divided into six groups of similar average body weight (*n* = 8–10 per group). The generation of *Ffar3*^−/−^ and *Ffar2*^−/−^ mice was described previously^36,40^.

The body weights were measured once a week. Food intake was calculated as the average of daily food intake (g/day per mouse) for 4 weeks. All mice were then sacrificed under deep isoflurane-induced anaesthesia, and the liver, caecum, epididymal, perirenal, and subcutaneous adipose tissues were harvested and weighed. Blood was collected from the inferior vena cava using heparinised tubes and plasma was separated by immediate centrifugation (7,000 × *g*, 5 min, 4 °C). For intraperitoneal phosphate buffered saline (for control group) or propionate administration (sodium propionate, Wako, 1 g/kg body weight) as previous described^36^, 7-week-old wild-type and *Ffar3*^−/−^ mice on C57BL/6J background were sacrificed and collected samples at 24 h after propionate administration under HFD feeding. All tissues and plasma were stored at −80 °C until further processing. All experimental procedures involving mice were performed in 2017–2019 according to protocols approved by the Committee on the Ethics of Animal Experiments of the Tokyo University of Agriculture and Technology (Permit No. 28-87). All efforts were made to minimise suffering.

### Plasma biochemical analyses

The blood glucose concentration was measured using One Touch Ultra Test Strips (One Touch^®^ Ultra^®^, Life Scan, Milpitas, CA, USA). The concentrations of plasma total cholesterol (Lab Assay™ Cholesterol, Wako, Tokyo, Japan), NEFAs (Lab Assay™ NEFA, Wako, Tokyo, Japan), plasma and hepatic TG (Lab Assay™ Triglyceride, Wako, Tokyo, Japan), plasma PYY (Mouse/Rat PYY ELISA Kit, Wako, Tokyo, Japan), GLP-1 (GLP-1 Active ELISA Kit, Merck Millipore, Darmstadt, Germany), and insulin (Insulin ELISA KIT (RTU), Shibayagi, Gunma, Japan) were measured following the manufacturer’s instructions. For plasma GLP-1 measurement, the plasma sample was treated with a dipeptidyl peptidase IV inhibitor (Merck Millipore, Darmstadt, Germany) to prevent the degradation of active GLP-1. SCFA levels in the plasma and caecum were determined following a modification of a previously described protocol^49^. Herein, the SCFA-containing ether layers were collected and pooled for gas chromatography-mass spectrometry analysis using a GCMS-QP2010 Ultra system (Shimadzu, Kyoto, Japan). The calibration curves for SCFAs were constructed, and the concentration of each SCFA in the samples was evaluated over a specified concentration range.

### GTT and ITT

For GTT, 16-h fasted mice were given 2.0 mg of glucose per gram of body weight intraperitoneally (i.p.). For ITT, 3-h fasted mice were given human insulin (0.75mUg^−1^, i.p., Sigma). The blood glucose concentration was measured before injection and monitored at 15, 30, 60, 90 and 120 min after injection.

### Quantification of hepatic TG

The liver contents were weighed and stored at −80 °C until further processing. Hepatic triacylglycerol contents were measured following a modification of a previously described protocol^40^. Briefly, liver homogenates were subjected to crude lipid extraction using a mixture of chloroform/methanol/0.45 M acetic acid. Subsequently, 3 volumes of mixture were added and shaken overnight (4 °C). After centrifugation at 1,500 × *g* for 10 min, the organic layer was collected, dried, and resuspended in isopropyl alcohol. Measurements were conducted using the Lab Assay™ Triglyceride (Wako, Tokyo, Japan).

### Hepatic histology

Livers were embedded in OCT compounds (SAKURA, Finetek, Japan) and sectioned at 10 μm. Hematoxylin–eosin and oil red O (Sigma) staining were based on a previously described protocol with modification^40^, and examined with fluorescence microscope (All-in-One Fluorescence Microscope BZ-X700, KEYENCE, Osaka, Japan).

### Analysis of gut microbiota by 16S rRNA gene sequencing

Faecal DNA was extracted using Fast DNA^®^ SPIN Kit (MP Biomedicals, Santa Ana, CA, USA), and the V4 region of the 16S rRNA gene was amplified using dual-indexed primers. The amplicons were then sequenced using an Illumina MiSeq with a MiSeq Reagent kit V3 (Illumina, San Diego, CA, USA). Paired-end sequencing was carried out using the Illumina MiSeq platform. Processing and quality filtering of the reads were performed with Quantitative Insights into Microbial Ecology (QIIME) (v1.9.1) software, and the chimera-free sequences were aligned with the SILVA database (http://www.arb-silva.de) at a 97% identity threshold.

### Real-time polymerase chain reaction (RT-PCR)

RT-PCR was conducted following a modification of a previously described protocol^40^. RT-PCR was completed with SYBR Premix Ex Taq II (TaKaRa, Shiga, JAPAN) and the Step One^TM^ Real-time PCR system (Applied Biosystems, Foster City, CA, USA). The ∆∆CT method was used to determine the relative expression levels with the mRNA levels of the housekeeping *18S* gene as reference. The primer sequences are shown in Supplementary Table S2.

### Statistical analysis

All values are presented as the mean ± SEM. The statistical significance of differences between groups was determined using a two-tailed unpaired Student’s *t*-test (two groups) or two-tailed one-way analysis of variance, followed by Dunnett’s post hoc test (≥three groups). To estimate the required sample size for each experiment, a priori power analysis was performed using the G*Power software ver3.1 (Franz Faul, Universiät Kiel, Germany; http://www.gpower.hhu.de/). Sample size used in the present study will achieve 95% actual power to detect an effect of each experiment assuming a one-way ANOVA with a 0.05 significance level. Except for the power analysis, all experimental data analyses were performed using GraphPad Prism 7.0 (Graphpad Software, San Diego, CA, USA).

## Supplementary information

Supplementary Info

## Data Availability

The raw 16S rRNA sequence data have been deposited into the DNA Data Bank of Japan (DDBJ) database under accession no. DRA008619 [https://ddbj.nig.ac.jp/DRASearch/submission?acc = DRA008619].
