# Supplementary material for: Dietary short-chain fatty acid intake improves the hepatic metabolic condition via FFAR3
Source: Sci Rep. 2019 Nov 12;9:16574. doi: 10.1038/s41598-019-53242-x (PMC6851370; doi:10.1038/s41598-019-53242-x)
Supplement: Supplementary file 1 — Supplementary Info [file 41598_2019_53242_MOESM1_ESM.pdf]

SUPPLEMENTARY INFORMATION

Dietary short-chain fatty acid intake improves the hepatic metabolic condition via FFAR3

Hidenori Shimizu[1,2,3], Yuki Masujima[1], Chihiro Ushiroda[1,2], Rina Mizushima[1], Satsuki Taira[1], Ryuji Ohue-Kitano[1,2], Ikuo Kimura[1,2,*]

[1]Department of Applied Biological Science, Graduate School of Agriculture, Tokyo University of Agriculture and Technology, Fuchu-shi, Tokyo 183-8509, Japan, [2]AMED-CREST, Japan Agency for Medical Research and Development, Chiyoda-ku, Tokyo 100-0004, Japan, [3]NOSTER Bio-Institute, Nitto Pharmaceutical Industries, Ltd., Kamiueno, Muko, Kyoto 617-0006, Japan.

***Correspondence and requests for materials should be addressed to I.K. (email: ikimura@cc.tuat.ac.jp**

This PDF file includes:

Supplementary Figures 1–6

Supplementary Table 1, 2

Supplementary Figure S1

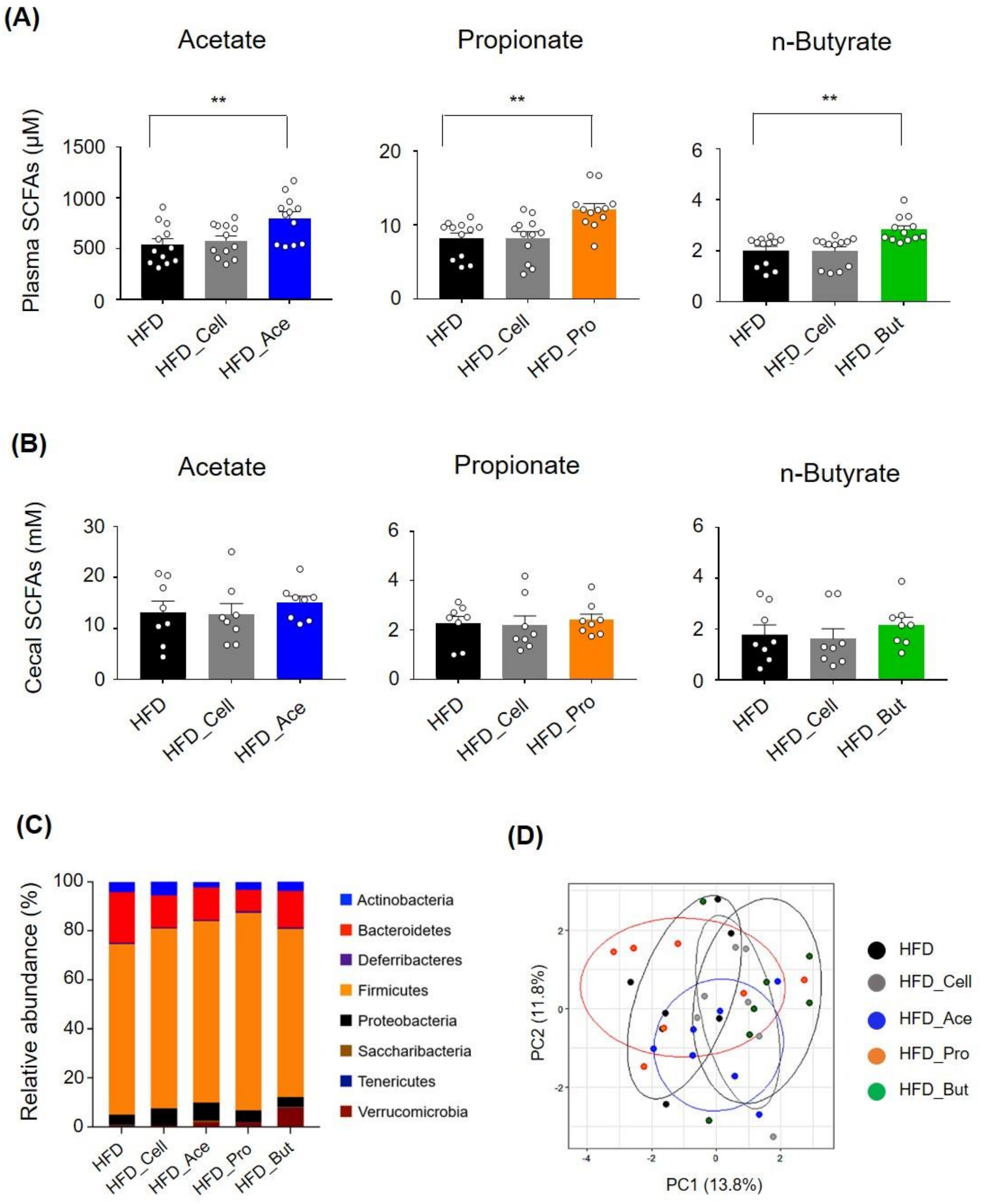

Supplementary Figure S1. Short-chain fatty acid (SCFA) supplementation increases plasma SCFA levels.

Plasma SCFAs (A), cecal SCFAs (B), and relative abundance (C) and composition as a PC2 plot (D) of faecal microbiota (phylum) measured after 4 weeks of high-fat diet (HFD) feeding with 5% cellulose, acetate, propionate, or butyrate. All data are presented as the means ± SEM (n = 7–12). Dunnett’s test; $^{**}P < 0.01$, compared with HFD.

Supplementary Figure S2

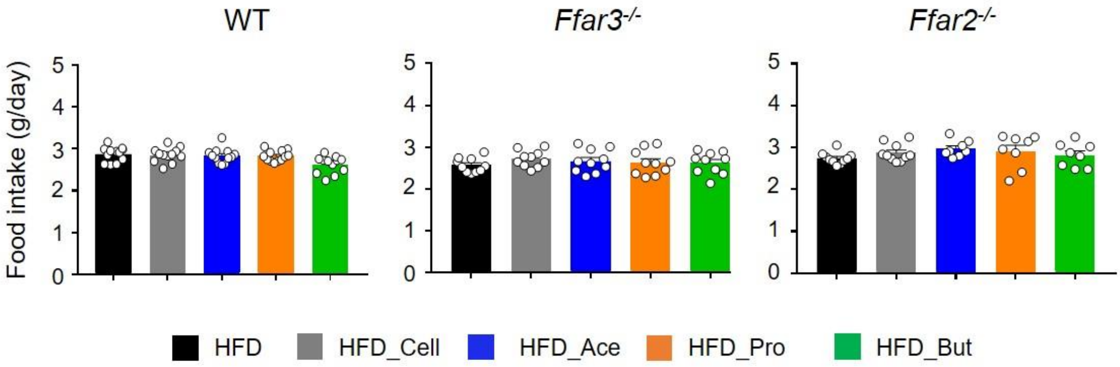

Supplementary Figure S2. Influence of high-fat diet (HFD) feeding supplemented with short-chain fatty acids (SCFAs) in *Ffar3*$^{-/-}$ and *Ffar2*$^{-/-}$ mice.

Food intake was measured after 4 weeks of HFD feeding supplemented with 5% SCFAs (*n* = 8–12). All data are presented as the means ± SEM

Supplementary Figure S3

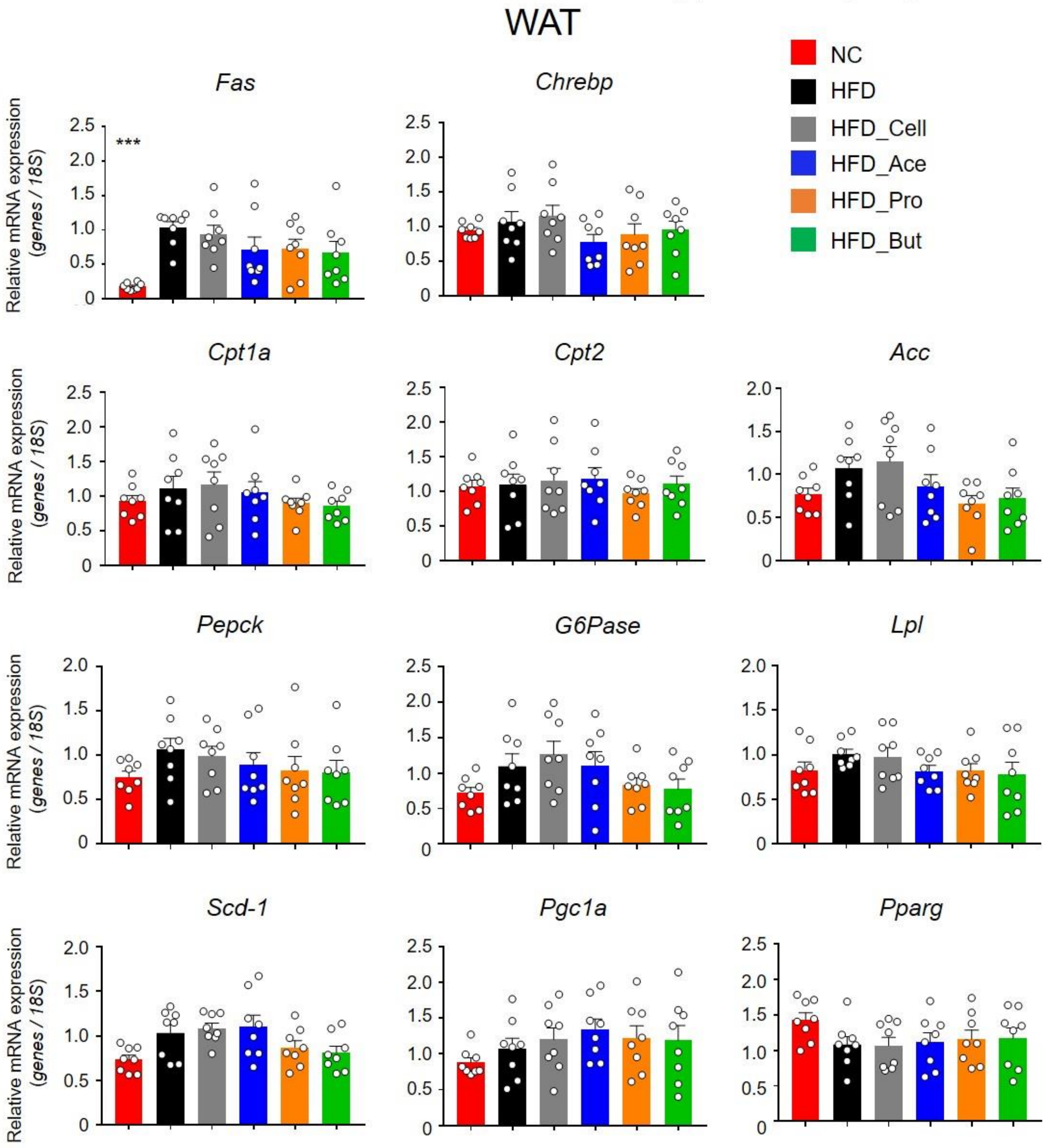

Supplementary Figure S3. mRNA expression profiles of energy metabolism-related genes in the white adipose tissue (WAT) under short-chain fatty acid (SCFA) feeding.

mRNA expression levels of energy metabolism-related genes in the WAT were measured after 4 weeks of control (NC) or high-fat diet (HFD) feeding supplemented with 5% SCFAs. All data are presented as the means ± SEM ($n = 8$). Statistical analysis was performed with Dunnett's test; ***$P < 0.001$, compared with HFD.

Supplementary Figure S4

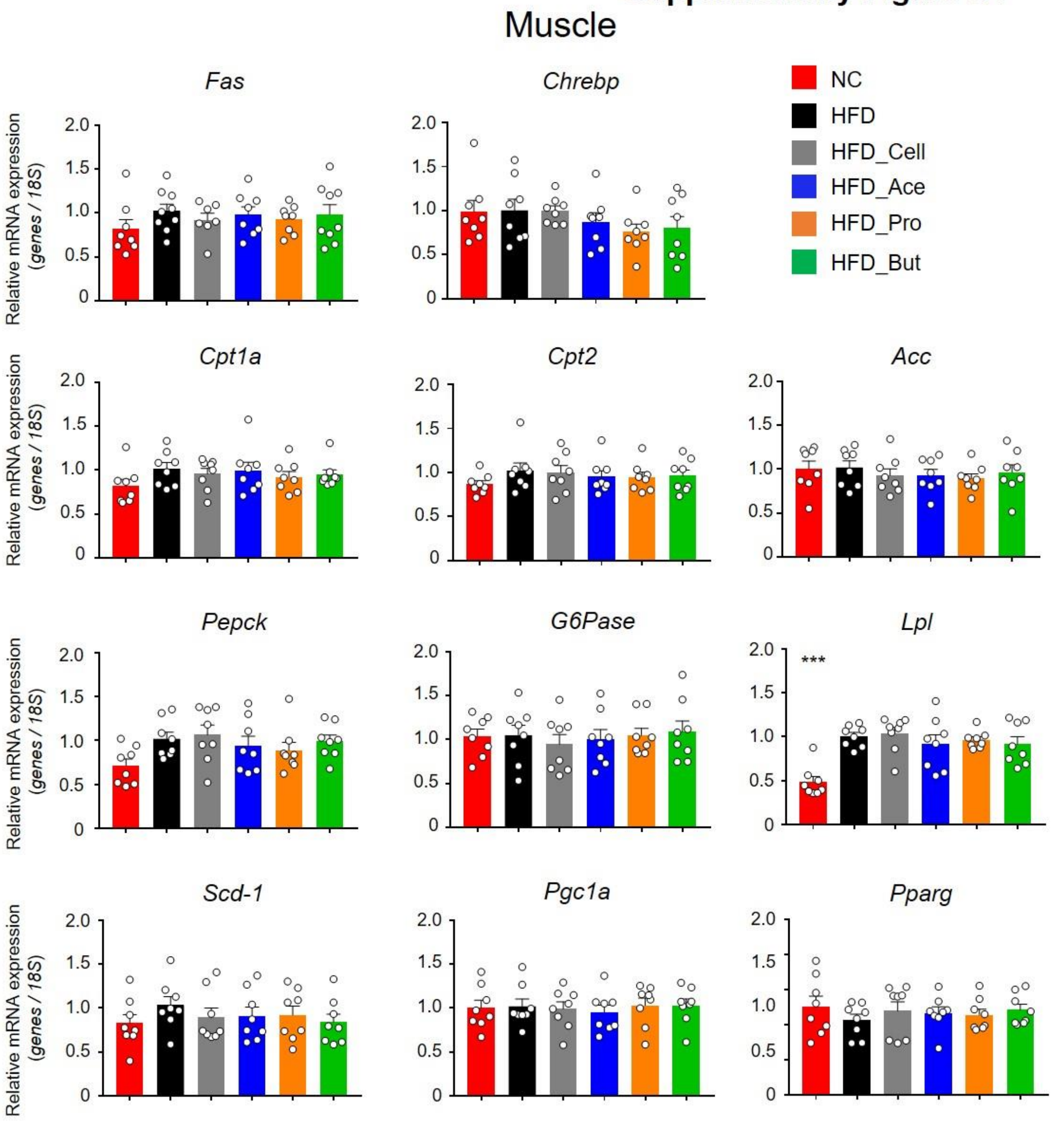

Supplementary Figure S4. mRNA expression profiles of energy metabolism-related genes in the muscle under short-chain fatty acid (SCFA) feeding.

mRNA expression levels of energy metabolism-related genes in the muscle were measured after 4 weeks of control (NC) or high-fat diet (HFD) feeding supplemented with 5% SCFAs. All data are presented as the means ± SEM (n = 8). Statistical analysis was performed with Dunnett's test; ***P < 0.001, compared with HFD.

Supplementary Figure S5

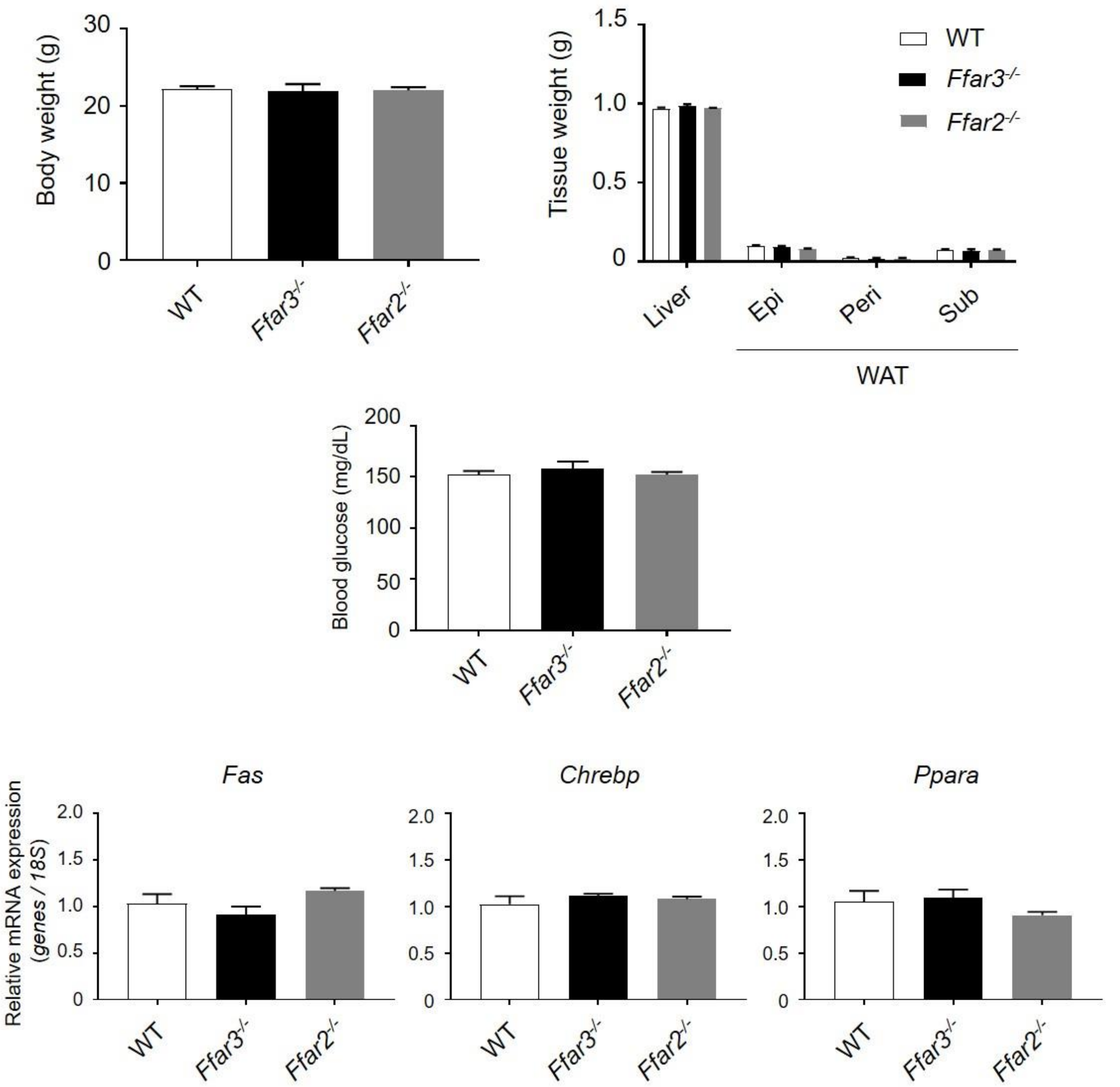

Supplementary Figure S5. Initial metabolic parameters in *Ffar3*$^{-/-}$ and *Ffar2*$^{-/-}$ mice.

Initial body, liver, white adipose tissue (WAT) weights, and blood glucose in 7-week-old wild-type (WT) (n = 8), *Ffar3*$^{-/-}$ and *Ffar2*$^{-/-}$ mice (n = 4) was measured before HFD feeding. All data are presented as the means ± SEM.

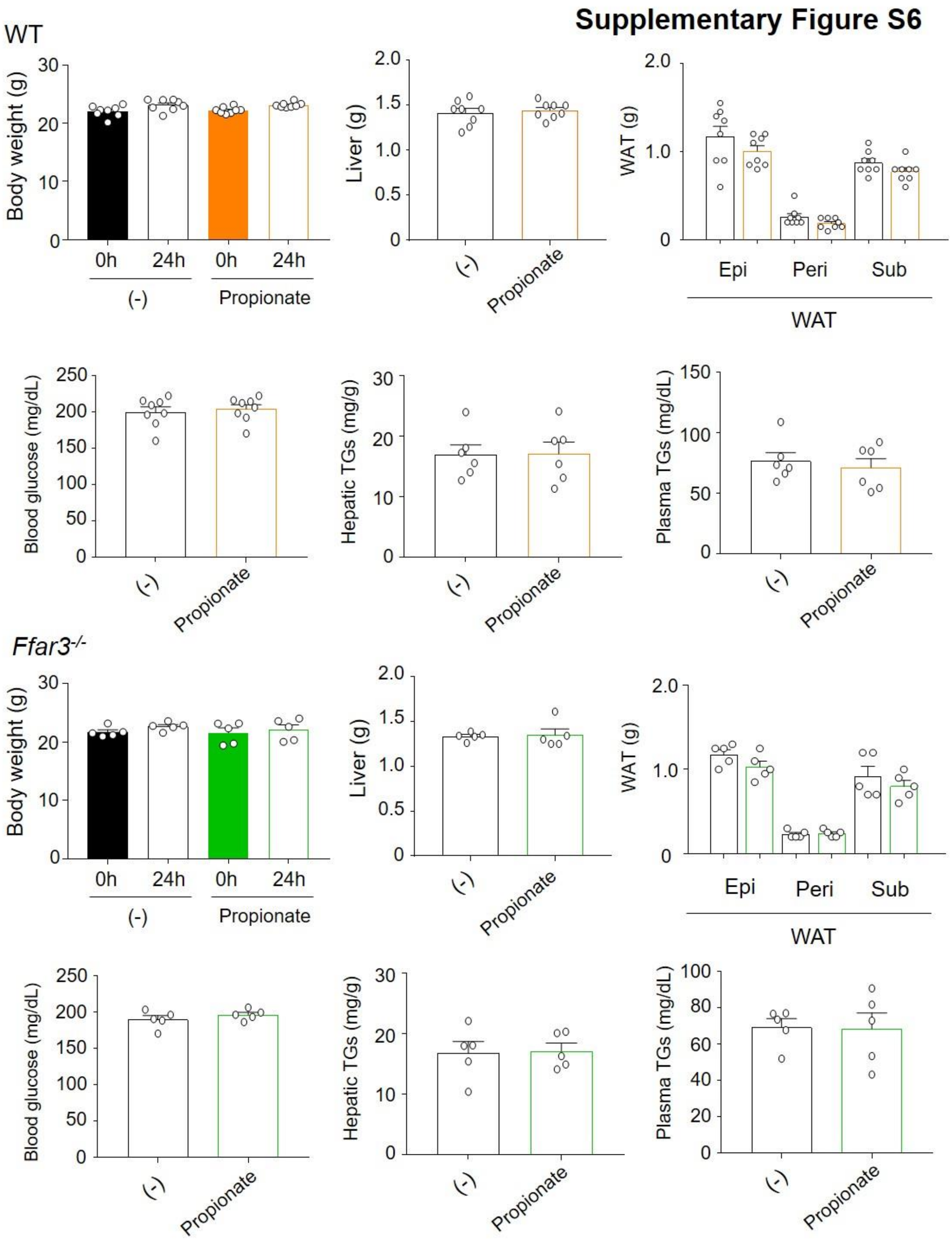

Supplementary Figure S6. Effects of acute propionate administration to *Ffar3*$^{-/-}$ mice.

Body weight changes, the weights of liver and white adipose tissues (WAT), blood glucose, hepatic triglycerides contents, and plasma triglyceride levels in wild-type (WT) and *Ffar3*$^{-/-}$ mice at 24 h after intraperitoneal phosphate buffered saline (for control group) or propionate administration (1 g/kg body weight) under high-fat diet (HFD) feeding. All data are presented as the means ± SEM (*n* = 5–8).

Supplementary Table S1

Formula	HFD [1]	Cellulose	Acetate	Propionate	Butyrate
Product			kcal %		
Protein	20	20	20	20	20
Carbohydrate	20	20	20	20	20
Fat	60	60	60	60	60
Ingredient			gm		
Casein, 30 mesh	200	190	190	190	190
L-cystine	3	2.85	2.85	2.85	2.85
Maltodextrin 10	125	118.75	118.75	118.75	118.75
Sucrose	68.8	65.36	65.36	65.36	65.36
Cellulose, BW200	50	47.5	47.5	47.5	47.5
Cellulose	0	38.6925	0	0	0
Acetate	0	0	38.6925	0	0
Propionate	0	0	0	38.6925	0
Butyrate	0	0	0	0	38.6925
Soybean oil	25	23.75	23.75	23.75	23.75
Lard	245	232.75	232.75	232.75	232.75
Mineral mix S10026	10	9.5	9.5	9.5	9.5
Dicalcium phosphate	13	12.35	12.35	12.35	12.35
Calcium carbonate	5.5	5.225	5.225	5.225	5.225
Potassium citrate, 1 H_2O	16.5	15.675	15.675	15.675	15.675
Vitamin mix V10001	10	9.5	9.5	9.5	9.5
Choline bitartrate	2	1.9	1.9	1.9	1.9
FD&C blue dye [#1]	0.05	0.0475	0.0475	0.0475	0.0475

[1] HFD: high-fat diet
FD&C blue dye[#1]: synthetic organic compound primarily used as a blue colorant for dietary supplements.

Supplementary Table S1. Composition of high-fat diets.

Supplementary Table S2

Gene	Forward	Reverse
18s	5'-ACGCTGAGCCAGTCAGTGTA-3'	5'-CTTAGAGGGACAAGTGGCG-3'
Fas	5'-GCTGCGGAAACTTCAGGAAAT-3'	5'-AGAGACGTGTCACTCCTGGACTT-3'
Acc	5'-AAGGCTATGTGAAGGATG-3'	5'-CTGTCTGAAGAGGTTAGG-3'
Chrebp	5'-CTGGGGACCTAAACAGGAGC-3'	5'-GAAGCCACCCTATAGCTCCC-3'
Cpt1a	5'-GCATAAACGCAGAGCATTCC-3'	5'-GATGTTGGGGTTCTTGTCTCC-3'
Cpt2	5'-CTCATCCGCTTTGTTCCTTC-3'	5'-AGTTCATCACGACTGGGTTTG-3'
Ppara	5'-CCTGAACATCGAGTGTCGAA-3'	5'-GGCCTTGACCTTGTTCATGT-3'
Pepck	5'-CCACAGCTGGTGCAGAACA-3'	5'-GAAGGGTCGATGGCAAA-3'
G6Pase	5'-CCATGCAAAGGACTAGGAACAA-3'	5'-TACCAGGGCCGATGTCAAC-3'
Lpl	5'-CTGCTGGCGTAGCAGGAAGT-3'	5'-GCTGGAAAGTGCCTCCATTG-3'
Scd-1	5'-GTCAGGAGGGCAGGTTTC-3'	5'-GAGCGTGGACTTCGGTTC-3'
Pgc1a	5'-GAGAATGAGGCAAACTTGCTAGCG-3'	5'-TGCATGGTTCTGAGTGCTAAGACC-3'
Pparg	5'-TCAGCTCTGTGGACCTCTCC-3'	5'-ACCCTTGCATCCTTCACAAG-3'

Supplementary Table S2. Primer sequences used in this study.
